# Breast cancer trends in Chile: Incidence and mortality rates (2007–2018)

**DOI:** 10.1371/journal.pgph.0001322

**Published:** 2024-06-27

**Authors:** Benjamín Madariaga, Susana Mondschein, Soledad Torres

**Affiliations:** 1 Department of Mathematical Engineering, University of Chile, Santiago, Chile; 2 Department of Industrial Engineering, University of Chile, Santiago, Chile; 3 Instituto Sistemas Complejos de Ingeniería (ISCI), Santiago, Chile; 4 Center for Cancer Prevention and Control (CECAN), Santiago, Chile; 5 Centro Integral de la Mama, Clínica Las Condes, Santiago, Chile; PLOS: Public Library of Science, UNITED STATES

## Abstract

Breast cancer (BC) is one of the most common cancers in women worldwide and in Chile. Due to the lack of a Chilean national cancer registry, there is partial information on the status of BC in the country. We aim to estimate BC incidence and mortality rates by health care providers and regions for Chilean women. We used two public anonymized databases provided by the Ministry of Health: the national death and hospital discharges datasets. We considered a cohort of 58,254 and 16,615 BC hospital discharges and deaths for the period 2007–2018. New BC cases increased by 43.6%, from 3,785 in 2007 to 5,435 in 2018. Total BC deaths increased by 33.6% from 1,158 to 1,547 during the same period. Age-adjusted incidence rates were stable over time, with an average rate of 44.0 cases/100,000 women (SD 2.2). There were considerable differences in age-adjusted incidence rates among regions, with no clear geographical trend. Women affiliated to a private provider (ISAPRE) have an average age-adjusted incidence rate of 60.6 compared to 38.8 (both cases/100,000 women) for women affiliated with the public provider (FONASA). Age-adjusted mortality rates have an average of 10.5 cases/100,000 women (SD 0.4). This study shows important differences in incidence rates between private and publicly insured women, with no significant differences in mortality rates. Such differences may be associated with women’s lifestyles, dietary compositions, comorbidities, and differences in healthcare systems. These hypotheses should be studied in greater depth. Additionally, differences in BC incidence found in this study compared to incidences reported from other estimations reinforce the need of a national cancer registry that should lead to more accurate indicators regarding BC in Chile.

## 1 Introduction

Breast cancer (BC) is one of the most common cancers in women worldwide, with 2.26 million new cases and 685 thousand deaths globally in 2020 and 7.8 million women alive with breast cancer diagnosed in the past 5 years [1]. In 2020, age-standardized world breast cancer incidence and mortality rates were 47.8/100,000 and 13.6/100,000 women, respectively [2], while the figures reported for Chile by the Ministry of Health (Minsal) for 2018 were 44.1/100,000 and 11.3/100,000 women for incidence and mortality rates, respectively [3, 4].

Unfortunately, in Chile, there is no national cancer registry, and thus, incidence rates have been estimated based on projections on the number of breast cancer cases diagnosed from 1998 to 2012, with follow-ups until 2015, from four regional population-based registries (Antofagasta and Los Ríos regions and Concepción and Biobío provinces). The estimates for the rest of the country and from 2013 onward are based on statistical models, with assumptions such as a constant fatality rate across regions and over time, and in some instances, using neighboring country data [3, 5]. However, these assumptions might lead to unreliable estimates when ignoring significant differences in ethnicity, urbanization, socioeconomic composition, and health coverage among regions [6–9] and advances in breast cancer treatment over time. Thus, for example, fatality rates might largely vary among regions due to differences in incidence as well as in mortality rates and over time due to a decrease in mortality rates.

On the other hand, mortality rate estimates are much more reliable because of the existence of a high-quality national death registry that includes a series of demographic variables along with the cause of death of every decedent in Chile. The death registry has been used in studies such as those by Icaza, Nuñez, and Bugueño [10], where the authors exploit the potential of this registry by studying breast cancer mortality, including ecological analysis by sociodemographic variables.

The Chilean health care system consists of a hybrid of public and private insurance plans as well as providers. The three main insurance systems are the National Health Fund (FONASA) for 78% of the Chilean population, private health care insurers (ISAPREs) for 14% of the Chilean population, and the Military and Police Forces’ health system for 3% of the Chilean population. The remaining 5% of the population either has no health care insurer or their insurance status is unknown. Privately insured patients can solely access private providers, with a variety of coverage, depending on their health care plan and monthly payments. On the other hand, FONASA patients–paying, on average, a lower monthly price compared to those in the private system, might access public and private hospitals, depending on the health care plan within FONASA, with different amounts of copayments. Thus, the FONASA has four different segments of patients (A, B, C, and D), with A and D being those plans with the lowest- and highest-income patients, respectively. FONASA also provides coverage for attending private health centers to patients from the B, C, and D segments [11].

The selection of the health care provider is determined mainly based on the individual’s economic income [12, 13]; as a consequence, profound inequalities in terms of quality of life and education, among others, have existed between users of the private and public affiliates. Health care indicators have been significantly better for those with private health insurance compared to those for publicly insured patients. For example, in 2017, 27.8% of FONASA affiliates declared having problems obtaining health care, while this percentage decreased to 12.6% for ISAPRE affiliates [6].

The goal of this paper is the estimation of breast cancer incidence and mortality rates, at a national and regional levels using an alternative methodology, based on a complete anonymized public database of national registry of hospital discharges, which includes information and diagnosis at the patient level, and the national death registry, with primary and secondary causes of death through a unique ID classification, compiled by the Department of Health Statistics and Information (DEIS) of the Ministry of Health. We also compute incidence and mortality by healthcare insurance system.

## 2 Methods and materials

### 2.1. Ethics statement

This work used publicly available data from the Chilean Ministry of Health through the Department of Health Statistics and Information. All data were protected, and personal information was anonymized; therefore, no consent from participants was needed.

### 2.2 Data

Two public anonymized databases were provided by DEIS. The first is the National Death Registry, which includes 2,549,800 deaths from January 1990 to December 2018. For each death entry in the registry, the patient’s ID (identifying code), date of birth, date of death, gender, town and region of residence, marital status, occupation and cause of death code according to the International Statistical Classification of Diseases and Related Health Problems (CDI-10) were available. There were 2,984 death registries without an ID, which corresponds to 0.1% of the complete database. The second database includes all discharges from public and private health care facilities in the country, which consists of 32,443,591 registries from January 2001 to December 2020. Each registry has 39 fields, such as the patient’s ID (same as the national death database), date of birth, gender, town and region of residence, ethnicity, health insurance, length of stay, condition at discharge, and primary and secondary diagnoses according to CDI-10 classification, among others.

It is important to emphasize that this internal ID created by the Department of Health Statistics and Information (DEIS) of the Ministry of Health allows to know if a patient that has been hospitalized has died or not within the study period and/or has been hospitalized multiple times and it is not possible to identify patients with it.

### 2.3. Inclusion and exclusion criteria

The cohort for our study corresponds to all women who were diagnosed with breast cancer from 2007 to 2018. First, we chose 2007 as the starting year to guarantee that the first discharge in the database is, in fact, the time of diagnosis.

Thus, all women with breast cancer discharges from 2007 onward, but none from 2001 to 2007, were included as newly diagnosed patients. Second, we did not include data from 2019 onward because it is not reliable due to the political and social uprising in Chile (October-December 2019) [14] and the COVID-19 pandemic, which largely influenced hospital discharges and deaths. Accordingly, we considered the same period of time for the national death registry.

A discharge and death registry is considered to correspond to breast cancer if its CDI-10 diagnostic code belongs to the categories C50 and D05 (*malignant neoplasm of breast* and *carcinoma in situ of breast*, respectively).

For calculations of mortality rates, we selected all females whose cause of death was directly associated with breast cancer from the 2,549,800 registries in the mortality database during the study period. For calculations of incidence rates, we selected all female patients whose primary diagnosis was breast cancer related from the 32,443,591 hospital discharges during the studied period. Some considerations were taken to assess missing data and inconsistencies, such as removing records with missing IDs. The Results section presents a sensitivity analysis of these considerations.

S1 supporting information provides further details on the inclusion and exclusion criteria for the death and discharge databases.

### 2.4 Methods

We calculated both crude and age–adjusted incidence and mortality rates for breast cancer at the national and regional levels. Population estimates and projections provided by the Chilean National Institute of Statistics (INE) [15, 16] were used. For the age-standardized incidence and mortality rates, we used the International Agency for Research on Cancer standard population [17]. We also calculated crude and age-standardized incidence and mortality rates by year, health insurer (public and private systems), and geographical region.

The female population affiliated with the public health system was obtained using the data provided by the FONASA in its yearly statistical bulletin, which is available online from 2009 onward [18]. Prior to this, FONASA bulletins had incomplete information, without segmentation by age intervals or gender. Therefore, we used linear regression to estimate the 2007 and 2008 populations from the available data.

Information from the private health system beneficiary population was obtained using the data provided by the Health Superintendence in its yearly statistics publication for ISAPRE’s beneficiaries [19].

## 3 Results

The resulting death database has 16,615 registries, meanwhile the resulting discharges database has 99,554 records corresponding to 58,254 patients, with an average of 1.67 discharges per patient.

Table 1 shows the number of deaths, average age of death, and standard deviation by year. The last two columns show the percentage of deaths that do not have a corresponding breast cancer discharge registry, this is, these patients died without hospitalization due to breast cancer, and their average age at death with standard deviation by year. Table 2 shows the number of newly diagnosed breast cancer cases, with the average age of diagnosis and standard deviation. It is important to remark that this table shows the discharges selected after applying the inclusion-exclusion criteria, and does not consider the 3,839 women with a cancer decease and without a hospital discharge.

**Table 1:** Total breast cancer deaths and percentage of breast cancer deaths without breast cancer discharge by year for the period 2007–2018.

	Total Deaths		Deaths without discharge*	
Year	Cases	Mean Age (std)	Cases(%)	Mean Age (std)
2007	1,158	65.4 (15.6)	28.2	73.6 (14.8)
2008	1,228	65.3 (15.6)	29.2	72.8 (15.1)
2009	1,337	65.8 (15.6)	24.8	75.5 (15.0)
2010	1,298	64.9 (15.6)	24.3	73.8 (15.3)
2011	1,349	66.2 (15.5)	22.4	75.2 (15.0)
2012	1,371	66.4 (15.6)	23.2	74.9 (15.2)
2013	1,391	65.8 (15.6)	24.5	75.9 (14.7)
2014	1,424	66.3 (16.0)	22.9	75.9 (15.2)
2015	1,512	66.5 (15.6)	23.1	76.5 (14.4)
2016	1,492	66.4 (15.3)	21.2	76.4 (14.7)
2017	1,508	66.8 (15.3)	24.3	76.7 (14.9)
2018	1,547	67.1 (16.0)	23.3	77.0 (14.5)
Total	16,615	66.1 (15.6)	24.1	75.4 (14.9)

* Women died due to breast cancer, but there were no hospital discharges associated with a breast cancer diagnosis. For the calculation of incidence, it is assumed that these women had a random uniform survival period between 1 and 12 months.

**Table 2:** New breast cancer cases by year for 2007–2018.

Year	New cases	Mean Age (std)
2007	3,785	57.4 (14.1)
2008	3,938	57.5 (14.1)
2009	4,576	57.3 (13.9)
2010	4,415	57.9 (14.1)
2011	4,685	57.7 (14.0)
2012	4,995	57.3 (13.8)
2013	5,172	57.7 (13.7)
2014	4,932	58.1 (13.7)
2015	5,427	57.8 (13.6)
2016	5,569	57.5 (13.8)
2017	5,325	58.1 (14.0)
2018	5,435	58.1 (13.9)
Total	58,254	57.7 (13.9)

The number of new breast cancer diagnoses increased by 43.6%, from 3,785 in 2007 to 5,435 in 2018. Total breast cancer deaths increased by 33.6% from 1,158 to 1,547 during the same period of time. We noticed a slight increasing trend in the mean age at death, with a 2.6% growth from 65.4 to 67.1, while there was no significant change in the mean age at diagnosis. We noted that the female population in Chile grew 13.3% from 8,390,194 to 9,506,921 during the period 2007–2018. In what follows, the results of incidence and mortality rates are shown.

### 3.1 Incidence rates

**Fig 1:**
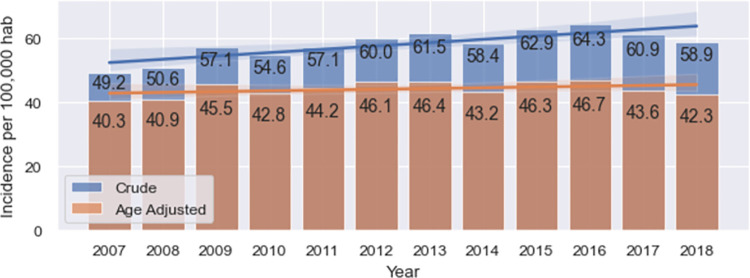
Crude and age-adjusted incidence rates by year (cases/100,000 women).

As explained in Subsection 2.2, incidence rates are calculated based on a set of 62,093 patients. In total, 58,254 patients were identified through the discharge registries plus the 3,839 breast cancer death registries without a discharge registry. An imputed incidence was only used for such cases. Fig 1 shows both crude and age-adjusted incidence rates (cases/100,000 women). We observe that the crude incidence rates are higher than the age-adjusted rates and have a growing trend over time, with an increase of 19.7%, from 49.2 in 2007 to 58.9 in 2018. The age-adjusted rates are stable over time, with an average rate of 44.0 and a standard deviation of 2.2 during the study period.

It is important to note that there were 3,371 discharge registries that were deleted for having no ID and, therefore, it cannot be determined if these registries correspond to patients who have been already identified in the cohort or if they correspond to new diagnoses. To evaluate the impact of these cases on the incidence estimates, we considered two possible scenarios. First, we considered the worst-case scenario, where all 3,371 registries are new breast cancer diagnoses. Table 3 shows the crude incidence rates calculated, accounting for each of these registries as a new diagnosis. Table 4 shows a more likely scenario, where some of these discharges do not belong to a new patient. Women from the resulting database had an average of 1.67 hospital discharges; then, as a more likely scenario, we considered that there is a newly diagnosed patient for every 1.67 discharges with missing IDs.

**Table 3:** Registries with missing IDs and crude incidence rates (cases/100,000 women) calculated considering these as new cases (worst case scenario). The last row shows the variation between these crude rates and those shown in Fig 1.

Year	2007	2008	2009	2010	2011	2012	2013	2014	2015	2016	2017	2018
Registries with missing IDs	1286	528	276	241	225	245	206	164	28	28	47	97
Crude Incidence	64.5	56.8	60.4	57.4	59.7	62.7	63.8	60.2	63.2	64.6	61.4	59.9
Variation (%)	23.8	11.0	5.3	4.8	4.3	4.4	3.6	3.0	0.5	0.5	0.8	1.7

**Table 4:** Registries with missing IDs and crude incidence rates (cases/100,000 women) calculated considering only a portion of these as new cases (likely scenario). This equals one new diagnosis every 1.67 discharges. The last row shows the variation among these crude rates and those shown in Fig 1.

Year	2007	2008	2009	2010	2011	2012	2013	2014	2015	2016	2017	2018
Registries with missing IDs	772	317	166	145	135	147	124	98	17	17	28	58
Crude Incidence	58.4	54.3	59.1	56.3	58.6	61.6	62.9	59.5	63.1	64.4	61.2	59.5
Variation (%)	15.8	6.9	3.3	3.0	2.6	2.7	2.2	1.8	0.3	0.3	0.5	1.0

There was a large number of registries with missing IDs in 2007, accounting for more than a third of all registries with missing IDs. The years prior to 2007 also show a large number of discharges with missing IDs, with a yearly average of 1,820 for the 2001–2006 period. This may be due to registration errors during the first years that were corrected, resulting in a much lower number of missing IDs in the later years of the period.

As expected, the crude incidence rates obtained in both cases are higher than those shown in Fig 1, with the highest differences in 2007 and 2008. In the worst-case scenario, apart from these two years, all variations are lower than 5.3%. The mean crude rate from 2007–2018 was 61.2/100,000 women, with a standard deviation of 2.6. The mean crude incidence rate previously found is 57.9/100,000 women, with a variation between the two crude rates of 5.3%, which represents an upper bound considering the worst-case scenario for the error on the estimates shown in Fig 1. In the more likely scenario shown in Table 4, in addition to 2007 and 2008, all crude rates have a variation lower than 3.3%. The mean crude rate from 2007–2018 was 59.9/100,000 women, with a standard deviation of 2.9. This represents a variation with respect to the mean crude rate found in Fig 1 of 3.3%, which represents an upper bound considering a more likely scenario for the error.

**Fig 2:**
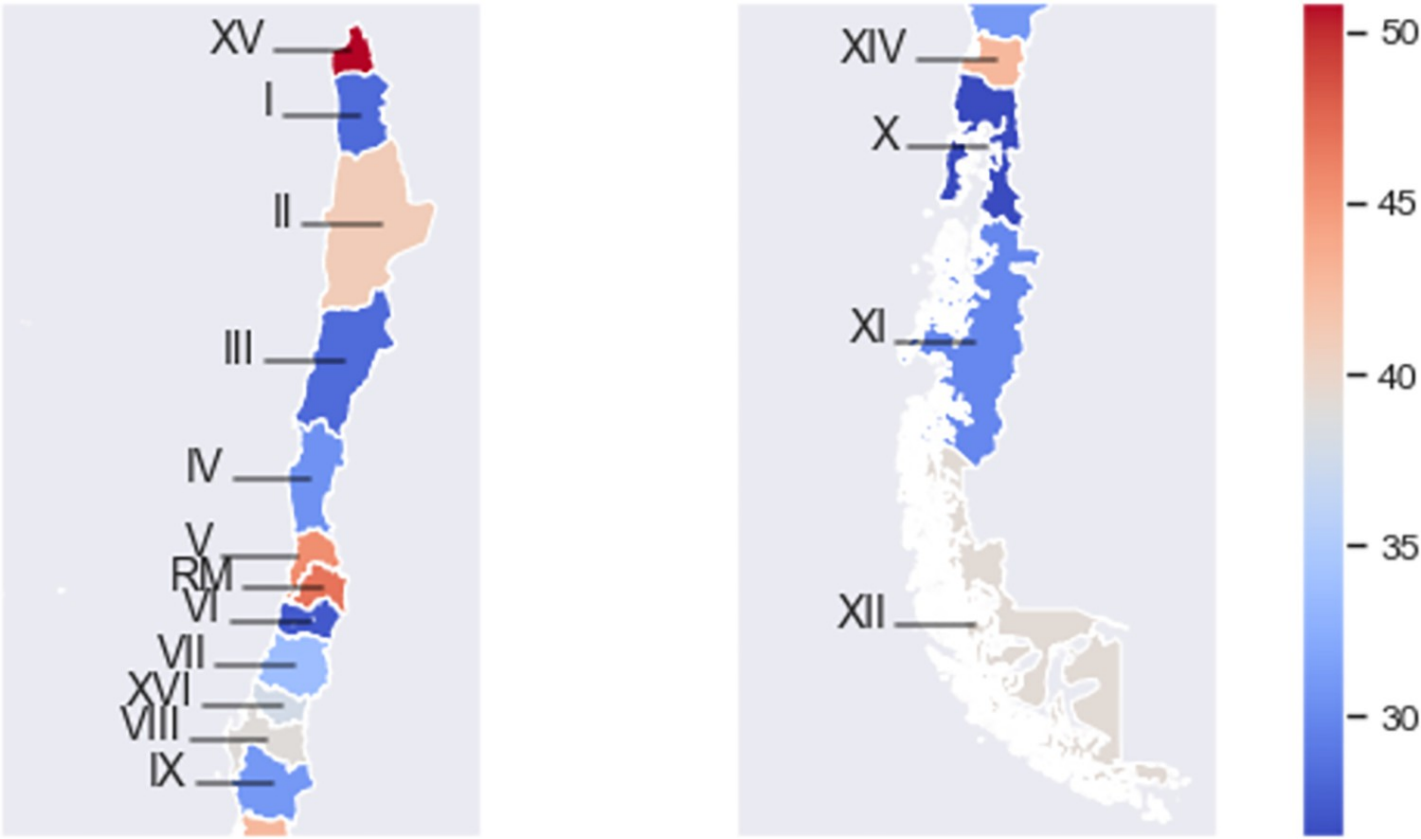
Mean age-adjusted incidence rates (cases/100,000 women) during 2007–2018 by geographical region. Detailed incidence values can be found in Table A in S3 Text. Shapefiles by region obtained from Chile’s National Congress Library (https://www.bcn.cl/siit/mapas_vectoriales/index_html).

There are considerable differences in age-adjusted incidence rates among regions, as shown in Fig 2, with no clear geographical trend. Higher incidence rates are found in the northern part of the country, the Arica and Parinacota region (XV), and in the central part of the country, corresponding to the Metropolitan region (RM) and Valparaiso region (V), with age-standardized incidence rates of 50.9, 46.8 and 45.5, respectively. Interestingly, two of the regions with the lowest rates were also found in the northern area, the Atacama (III) and Tarapacá (I) regions, with incidences of 28.2 and 28.3, respectively, surpassed only by a southern and a central region, Los Lagos (X) and O’Higgins (VI), with age-adjusted incidence rates of 26.5 and 27.3, respectively. The rest of the regions have an age-adjusted rate between 30.0 and 42.7.

Table 5 shows crude and age-adjusted incidence rates by year for women affiliated with the private and public health systems. For this analysis, 56,250 out of the 62,093 registries were considered, i.e., a total of 90.6% of all diagnosed women in the 2007–2018 period had an affiliation with the private and public health systems. Of the remaining 5,843 women, 1,944 women had armed forces health insurance, 885 women had no health insurance, and 3,014 women had missing information.

**Table 5:** Crude and age-adjusted incidence rates (cases/100,000 women) for women with private and public health insurance.

	ISAPRE		FONASA	
Year	Crude	Age adjusted	Crude	Age adjusted
2007	36.3	37.2	46.7	36.7
2008	41.5	41.9	49.1	37.9
2009	72.3	71.1	54.9	42.9
2010	66.0	65.3	50.5	37.4
2011	61.1	58.4	54.2	39.2
2012	64.2	61.2	56.4	40.6
2013	63.1	59.2	57.8	40.6
2014	71.3	66.5	53.3	36.5
2015	72.9	67.0	59.1	40.7
2016	84.0	75.1	57.6	39.3
2017	73.0	62.8	55.2	37.5
2018	72.3	61.2	53.6	36.7
Mean (Std)	64.8 (13.6)	60.6 (11.0)	54.0 (3.7)	38.8 (2.0)

We observed that women affiliated with a private provider (ISAPRE) had higher incidence rates during the period under study, having an average age-adjusted incidence rate of 60.6/100,000 compared to 38.8/100,000 for women affiliated with the public provider (FONASA). The age-standardized incidence rate for the private health system shows greater fluctuations over time (sd of 11.0) than that for the public sector (sd of 2.0).

### 3.2 Mortality rates

**Fig 3:**
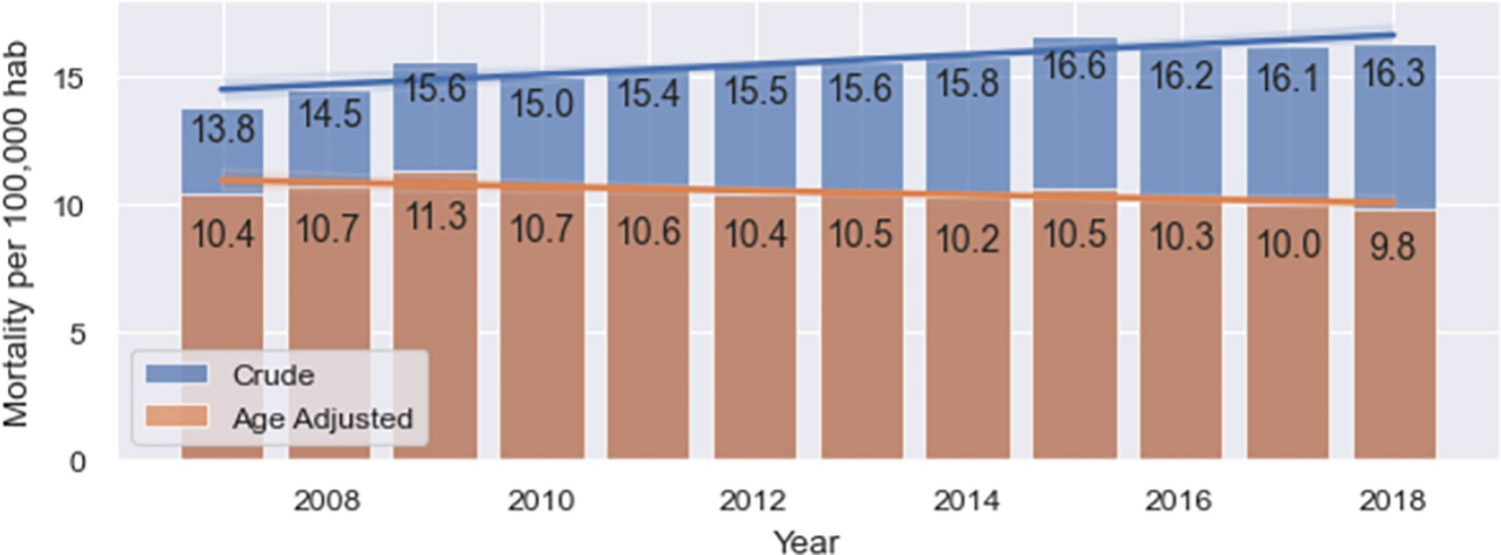
Crude and age-adjusted mortality rates (deaths/100,000 women) by year.

Fig 3 shows both crude and age-adjusted mortality rates (deaths/100,000 women) for the period under study. Crude mortality rates increased 18.1%, from 13.8 per 100,000 women in 2007 to 16.3 per 100,000 women in 2018. On the other hand, age-adjusted mortality rates did not have significant changes during the study period, with an average standardized mortality rate of 10.5 per 100,000 women and a standard deviation of 0.4.

**Fig 4:**
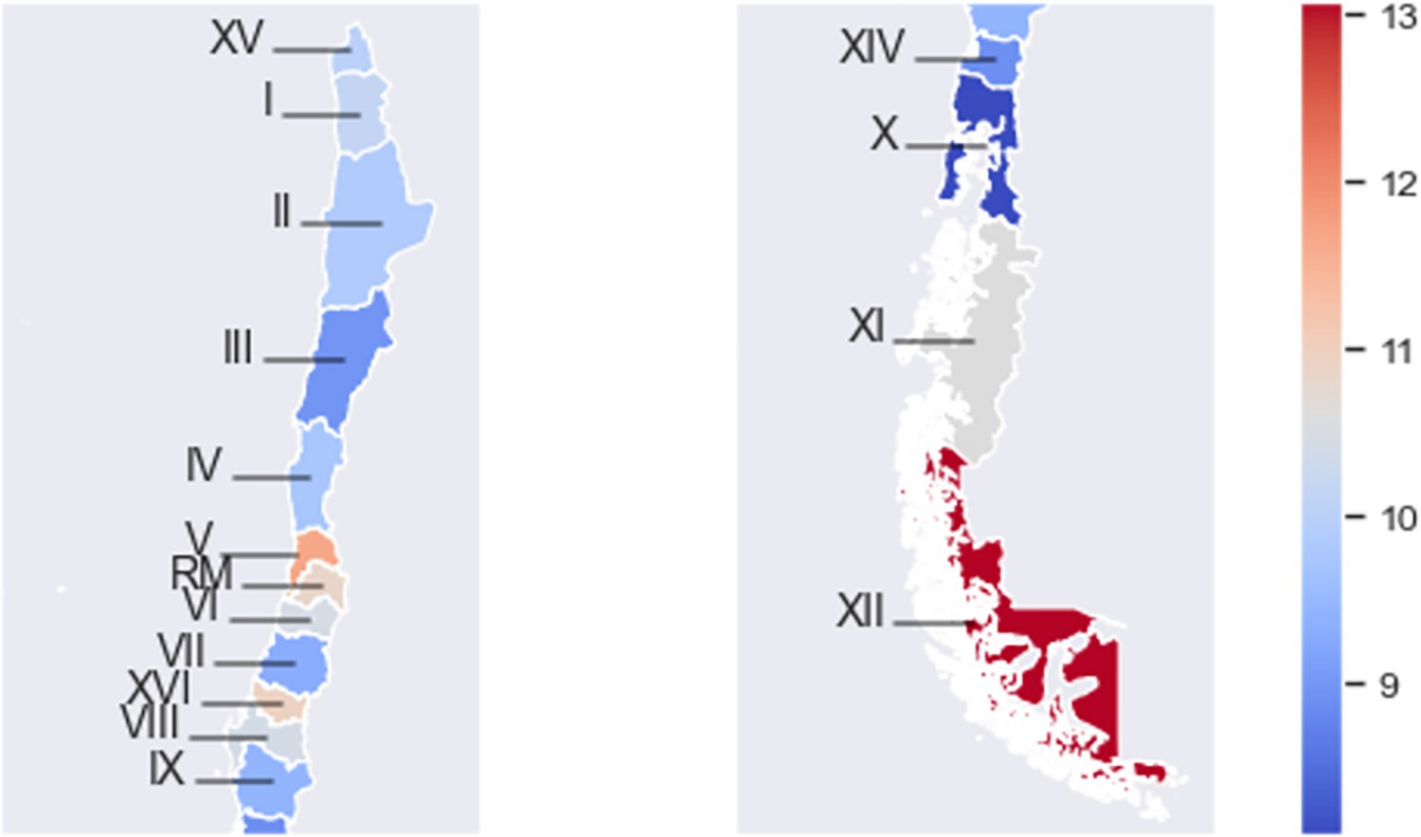
Mean age-adjusted mortality rates (deaths/100,000 women) during 2002–2018 by geographical region. Detailed mortality values can be found in Table A in S3 Text. Shapefiles by region obtained from Chile’s National Congress Library (https://www.bcn.cl/siit/mapas_vectoriales/index_html).

Fig 4 shows that the southern cities of Magallanes and Antártica Chilena (XII) have the highest age-adjusted mortality rate of 13.1, followed by the Valparaiso (V), Ñuble (XVI), and Metropolitan (RM) regions, with average age-adjusted rates of 11.7, 10.9, and 10.8, respectively. On the other hand, the regions with the lowest mortality rates are the southern regions of Los Lagos (X) and Los Ríos (XIV), with age-adjusted mortality rates of 8.1 and 8.9, respectively. The rest of the regions have similar mortality rates, averaging between 9.0 and 10.6 deaths per 100,000 women.

Table 6 shows mortality rates by year for women affiliated with the private and public health systems. As the national death registry does not include the health insurance of the deceased patients, we obtained them by searching the discharge database with the corresponding patient ID. Information was found for 89.3% of all women from the death database, corresponding to 14,837 women, though the rest did not have any hospital discharge data. From this set of women, 14,057 women belong to either the FONASA or ISAPRE. The last column of Table 6 shows the percentage of women from the death database from which it was not possible to recover their health care provider.

**Table 6:** Crude and age-adjusted mortality rates (deaths/100,000 women) for women with private and public health insurance. The last columns show the percentage of registries with no health insurance data.

	ISAPRE		FONASA		
Year	Crude	Age adjusted	Crude	Age adjusted	Missing (%)
2007	8.4	9.8	12.4	9.0	18.0
2008	8.0	8.6	13.0	9.2	16.9
2009	9.2	10.3	15.4	11.0	13.1
2010	9.4	10.2	13.9	9.5	13.3
2011	9.8	10.5	14.7	9.5	10.8
2012	8.7	8.8	15.0	9.3	9.5
2013	10.1	10.6	15.0	9.5	8.4
2014	9.7	10.1	15.5	9.4	8.7
2015	11.7	11.9	16.1	9.6	9.3
2016	10.3	9.8	16.1	9.6	7.7
2017	11.3	10.5	15.5	9.2	8.0
2018	11.1	9.8	15.5	8.9	8.3
Mean (Std)	9.8 (1.2)	10.1 (0.9)	14.8 (1.2)	9.5 (0.5)	10.7

We observe, in terms of crude rates, that FONASA patients have a higher mortality rate than ISAPRE patients, with rates of 14.8 and 9.8 per 100,000 women, respectively. When adjusted by age groups, FONASA rates decreased, while ISAPRE rates increased, resulting in FONASA patients having a slightly lower age-adjusted mortality rate of 9.5 compared to 10.1 for ISAPRE patients. Both systems had a rather constant trend in terms of age-standardized rates throughout the study period.

## 4 Discussion

Breast cancer is one of the two cancers, together with gallbladder cancer [20], that most frequently affect Chilean women. It is also the leading cause of death from cancer for Chilean women [21]. In 1995, the Cancer Unit of the Ministry of Health formed the National Breast Cancer Commission to focus on the design of a National Breast Cancer Program to better organize the care for breast diseases [22]. The commission defined referral, diagnostic, and treatment guidelines and carried out several activities across the country to improve breast cancer patients’ care.

As of 2004, a major reform in the Chilean health system was introduced: the Universal Access Plan for Explicit Guarantees (AUGE), later renamed GES, which aimed to provide timely and quality health care without discrimination and with financial protection to all Chilean residents. Thus, the Ministry of Health, through this plan, guarantees the coverage of a number of diseases (It started in 2005 with 25 pathologies and grew to 85 in 2022). Breast cancer was part of the initial pathologies included in 2005; in addition to the quality, access, financial, and opportunity guarantees, it also defined the complete protocol of medical treatments, including procedures and drugs to diagnose and treat breast cancer in all its stages. This protocol was updated in 2010 and 2015 according to the new treatment options that had been developed over time. In particular, in 2015, the high-cost drug trastuzumab was included for all Chilean women with HER2-positive breast cancer. The GES plan activates the above guarantees at the moment of a suspicion of breast cancer for people aged 15 years and over with suspected, diagnosed, or recurrent breast cancer [23]).

Once a patient detects a suspicious finding, either by self-detection or by routine mammogram and/or breast ultrasound, the patient is referred to a breast specialist physician who indicates a histologic confirmation, either by ultrasound-guided biopsy or by stereotaxic biopsy. Simultaneously, an immunohistochemical study is performed to determine tumoral markers. Depending on clinical presentation, a staging study may be requested immediately or after histological confirmation to rule out distant metastases. Once diagnostic work-up is completed, the case is discussed within a breast cancer board to determine the appropriate treatment.

Regarding early breast cancer detection, the Ministry of Health currently *recommends* an annual mammogram for women 50 to 69 years old (www.diprece.minsal.cl). However, despite this recommendation, it only guarantees triannual screening mammograms free of charge for all women 50 to 59 years old, independent of their health insurer [23]. The National Socioeconomic Characterization Survey, CASEN 2017 [6], shows that in 2017, only 53.4% and 33.1% of women aged 50 or more in the private and public health systems had a screening mammogram during the last year, respectively. These figures increase to 76.3% and 57.1% for a screening mammogram in the last three years.

These results might have changed with the implementation, as of July 2019, of a MINSAL strategy of increasing the coverage of mammograms in public primary health care, with important investments in new mammographers and the hiring of human resources in Family Health Centers (CESFAM) and community hospitals. One specific goal of this plan was to improve the coverage of breast cancer screening mammograms. For this purpose, the exams are taken by medical technologists in the aforementioned health care facilities and are sent electronically to the *mammography cell* (digital hospital), where the report is made by radiologists specialized in breast imaging, totaling 22% of the mammography reports in the country from January to September 2021 [24].

However, we note that in Chile there is no screening program as defined by the World Health Organization [25]. In particular, there is no information system in place linked to population registries that would allow the screenings to be offered to the eligible population using a call and recall system and have a registry of all screenings performed in the country.

With regard to epidemiological indicators, Chile does not have a national cancer registry. Hence, key estimates, such as incidence and case fatality rates, have been extrapolated from population records created in 1998 and updated until 2012 in the regions of Antofagasta, Biobío, Valdivia, and Concepción [26]. These regional data are insufficient to have a comprehensive view of the evolution of cancer at the national level due to differences in health care coverage, ethnicity, and regional particularities, among others. Furthermore, there is no reliable information from 2012 onward. Thus, we observe that most of the published research focuses on mortality indicators, of which there is a reliable national death database. However, regarding incidence rates, there are few studies with assumptions that might lead to inaccurate estimates. For example, the MINSAL study in 2019 [3] reported a constant drop in incidence rates, which is inconsistent with the data of the current state of breast cancer in Chile. This drop is obtained by the actual decrease in mortality rates and the assumption of a constant fatality rate over the study period, without considering the improvements in breast cancer treatments over time. These estimates feed the Chilean data in GLOBOCAN [5].

The incidence age-standardized rates found for the 2007–2018 period are lower than crude rates for the same period. This is largely explained by the demographic composition of the Chilean population. Breast cancer worldwide is significantly more common in older women and is less likely in women younger than 30 years old. This distribution is also observed in Chile. As Chile has a higher proportion of older women than the standardized population [15, 17], it is expected to have higher crude incidence rates. A similar result is found for mortality rates in the 2007–2018 period. The Global Cancer Observatory (GLOBOCAN) reported the Chilean incidence and mortality age-adjusted rates as 37.4 and 10.2 per 100,000 women in 2020, respectively [2]. Our study estimates incidence and mortality age-adjusted rates as 44.0 and 10.5 per 100,000 women, respectively, with a constant trend over the 2007–2008 period. We observe that our estimates of the mortality rate are consistent with those reported by GLOBOCAN, while our estimation of the incidence rate is higher than that of GLOBOCAN. The trends in age-standardized incidence reported by GLOBOCAN show that almost all countries have either had a constant trend (in countries such as Canada, Italy and Poland) or a growing trend (in countries such as Germany, Thailand or Korea) in the last 20 years. We remark that the constant trend found in our study for age-standardized incidence rates at the national level is consistent with this global trend observed by GLOBOCAN and differs from the decreasing trend found by the Ministry of Health [3]. It is important to note that the GLOBOCAN platform reports data on incidence and mortality based on information provided by countries and has no responsibility over their quality [5].

We also observe from Table 5 a much higher age-adjusted incidence rate in privately insured patients (ISAPRE) compared to those with public health care insurance. As discussed in the Introduction, these two groups of patients have important differences, such as income, lifestyle, diet composition, comorbidities, and use of hormonal therapies, among others, which might explain these incidence differences [27]. Another plausible explanatory hypothesis for this difference might rely on the higher use of spontaneous screening due to better access and awareness of its importance among privately insured women [6] and on the other hand, women who are public system beneficiaries are not well-informed about their right of having every 3 years a free mammogram between 50 to 59 years old, which should increase the detection at early stages and at early ages, or even in older women who otherwise would not be tested [28]. As previously stated, breast cancer is a disease covered by the GES program, and therefore, its diagnosis and treatment services are defined by law, hence these services must be delivered to every patient regardless of her healthcare provider. However, differences are observed in the waiting times for the first medical attention depending on the healthcare system type, being faster in the private system. Moreover, within the private system where patients may opt for GES modality or non-GES modality for their attention, the shortest waiting time is achieved in the latter. Such differences are related to the large gap that exists in the amount of population served in the public system compared to that in the private system, 80 versus 20%. In addition, public hospitals have become insufficient in relation to population growth. In terms of early detection, there is no national screening program. Nevertheless, there is a higher availability of mammograms in the private sector compared to those in the public sector: patients have a higher purchasing power and consequently, there are more private centers offering mammograms. Even some private clinics have opted to actively call their patients for annual breast screening exams. All of the above may be reflected in a higher number of diagnoses and earlier diagnoses in the private healthcare sector.

The estimated incidence and mortality rates shown by region in Fig 2 and Fig 4 show the great differences in breast cancer epidemiology along the Chilean territory. These differences indicate that the reality of each region must be taken into account when designing public health measures. Moreover, public measures and other related policies may be misdirected if they are designed and evaluated only through a few local registries, such as those from Antofagasta, Biobío, and Los Rios regions. This highlights the need for a national-level cancer registry from which adequate data can be obtained.

### 4.1. Study strengths and limitations

To the best of our knowledge, this is the first study on breast cancer incidence rates at the national level using publicly available data from several national registries on hospital discharges. Incidence has been reported by GLOBOCAN, estimating a 2020 incidence rate of 37.4. GLOBOCAN’s estimate is lower than the estimation of this study, and even more so considering the constant trend shown in Fig 1, with an average incidence of 42.3. Furthermore, this study highlights the important differences in key health care indicators for women across regions, ages, and health care insurance plans. The study of case fatality and survival rates for different segments of Chilean women is part of our ongoing research as well as the causes of these differences.

This study also has some limitations. First is the limitation of the quality of the national database used for hospital discharges and deaths. As discussed previously, there are missing and conflicting data and ambiguity in the cause of death in some death certificates. Furthermore, we used a hospital discharge database, and therefore, there might be some patients diagnosed with breast cancer who are never hospitalized when receiving treatment (for example, those who do not have surgery). Thus, incidence rates might be underestimated.

## 5 Conclusions

The methodology used in this study presents an alternative for obtaining incidence and mortality rates from public databases, which generates results consistent with other measures, especially in the absence of a national cancer registry, such is the case in Chile. We remark, however, that with the purpose of developing, implementing, and evaluating cancer public policies, it is of the utmost importance and priority to have a national cancer registry in Chile.

## Supporting information

S1 Text Appendix- inclusion and exclusion criteria. (DOCX)

S2 Text Appendix- other breast cancer diagnoses. (DOCX)

S3 Text Appendix- incidence and mortality results. (DOCX)

S1 Fig Inclusion and exclusion criteria for the breast cancer death database (2007–2018). (TIF)

S2 Fig Inclusion and exclusion criteria for the breast cancer hospital discharge database (2007–2018). (TIF)
