# Supplementary material for: Breast cancer trends in Chile: Incidence and mortality rates (2007–2018)
Source: PLOS Glob Public Health. 2024 Jun 27;4(6):e0001322. doi: 10.1371/journal.pgph.0001322 (PMC11210749; doi:10.1371/journal.pgph.0001322)
Supplement: S1 Text — (DOCX) [file pgph.0001322.s001.docx]

# S1 Appendix: Inclusion and exclusion criteria

Fig S1 shows details on the inclusion and exclusion of death registries. We selected all females whose cause of death was directly associated with breast cancer from the 2,549,800 registries in the mortality database, resulting in 16,615 BC deaths after removing 18 death registries with missing IDs, during the study period.

**Fig S1: Inclusion and exclusion criteria for the breast cancer death database (2007-2018).**

Fig S2 shows details on the inclusion and exclusion of discharge registries. We selected all patients whose primary diagnosis was breast cancer from the 32,443,591 hospital discharges, resulting in 154,379 discharges. There were 5,230 deaths that did not have a C50 and D05 discharge in the resulting database including 154,379 discharges. Thus, a set of diagnoses related to breast cancer was considered for such patients (see S2 appendix for a detailed explanation), leading to a total of 165,912 breast cancer-related discharges. Then, the cohorts from 2007 until 2018 were selected, resulting in 103,750 records corresponding to 58,914 patients. Out of the 103,750 discharge registries, there were 3,371 with a missing ID. Hence, we do not know if these discharges are associated with new patients or with those already in the cohort of 58,914 patients. Thus, discharge records with missing IDs were removed from the database. However, a sensitivity analysis regarding these registries is performed in the Results section. From the remaining 58,914 patients identified by their IDs, there were 987 patients who had inconsistencies in their gender, i.e., these patients had hospital discharges where they were identified both as male and female patients. These gender inconsistencies were corrected by considering the gender information from the mortality database and considering the mode of all records associated with such patients. Thus, the gender of 815 people was corrected in the discharges database, while 109 cases for which the mode was inconclusive were deleted.

Finally, all female patients were selected, resulting in a total of 99,554 discharge records, corresponding to 58,254 patients.

**Fig S2: Inclusion and exclusion criteria for the breast cancer hospital discharge database (2007-2018)**

* Several diagnoses were included considering health problems that could arise due to breast cancer progression or its treatment for patients who died due to breast cancer. See S2 appendix for further explanation.

** There were patients with records who identified them both as male and female. It was considered that this was an error from the database; therefore, it was corrected.
