# Supplementary material for: Breast cancer trends in Chile: Incidence and mortality rates (2007–2018)
Source: PLOS Glob Public Health. 2024 Jun 27;4(6):e0001322. doi: 10.1371/journal.pgph.0001322 (PMC11210749; doi:10.1371/journal.pgph.0001322)
Supplement: S2 Text — (DOCX) [file pgph.0001322.s002.docx]

# S2 Appendix: Other breast cancer diagnoses

After selecting discharges with a primary diagnosis code of breast cancer, there were still a considerable number of breast cancer death registries that did not have any associated discharge registry (5,230 deaths). However, there are discharge registries for causes other than breast cancer that are not typically included in a breast cancer incidence analysis. These are health problems that can arise due to the progression of the disease or its treatment (surgery, chemotherapy, radiotherapy) and therefore will be included under certain specific conditions. Thus, these diagnostics were taken into consideration only when they belonged to a patient who died because of breast cancer. We group these diagnostics into three groups:

1. Discharge registries directly attributable to breast cancer and its treatment. This item includes examination, treatment by chemotherapy and radiotherapy, and different breast-related diseases and issues, which may be due to breast cancer misdiagnosis. These diagnostics are D486, D24X, Z123, Z803, Z853, Z031, Z080, Z081, Z082, Z087, Z088, Z089, Z129, Z400, Z510, Z511, Z512, Z515, Z809, Z859, and Z860.
2. Diagnostics of other cancers and malignancies associated with breast cancer. This item includes secondary tumors and tumors of unspecified places. These diagnostics are C798, C782, C795, C793, C787, C786, C412, C800, D382, C792, C709, C383, C799, D059, C770, D420, C796, C414, C500, C413, C771, C728, C967, C779, D383, C399, C773, C781, C783, and C700.
3. Other diagnostics might be attributable to symptoms of breast cancer and its treatment. They are considered only if such discharge is close enough to the death registry. Each diagnosis has a different period of time to be associated with death and is shown in Table A. The absence of a period means that the diagnosis is always included independent of its gap from the death registry.

| Diagnostic Code Relation period | (years) |  | Diagnostic Code Relation period | (years) |
| --- | --- | --- | --- | --- |
| D649 | 2 |  | N850 | - |
| G039 | 1 |  | R060 | 1 |
| G540 | 2 |  | R17X | 1 |
| G939 | 1 |  | R18X | 1 |
| G952 | 2 |  | R51X | 1 |
| I495 | - |  | R53X | 1 |
| I891 | 1 |  | S220 | 4 |
| I972 | - |  | S320 | 2 |
| J80X | 2 |  | S323 | 4 |
| J90X | 2 |  | S325 | 4 |
| J948 | 2 |  | S327 | 4 |
| J960 | 2 |  | S328 | 4 |
| J969 | 1 |  | S423 | 4 |
| J984 | 2 |  | S720 | 4 |
| J989 | 2 |  | S721 | 4 |
| M532 | 4 |  | S722 | 4 |
| M544 | 4 |  | S723 | 4 |
| M546 | 4 |  | S724 | 4 |
| M549 | 4 |  | S728 | 4 |
| M808 | 4 |  | S729 | 4 |
| M844 | 4 |  | T08X | 4 |
| N63X | - |  | T12X | 4 |
| N645 | - |  | T142 | 4 |
| N648 | - |  | T932 | 4 |
| N649 | - |  |  |  |

**Table A**: CDI-10 codes and relation periods of the health problems to be considered as the breast cancer debut.

Through the addition of these diagnoses, 11,533 discharge registries were added to the discharge database, which corresponds to 1,219 patients. After the above process, there were still 3,839 patients with a death from breast cancer without a hospital discharge to estimate their incidence. For the calculation of incidence rates a random survival time between 1 and 12 months was considered for such women. A sensibility analysis was performed over such decisions, showing that the random period selected does not significantly affect the incidence rates.
