# Supplementary material for: Breast cancer trends in Chile: Incidence and mortality rates (2007–2018)
Source: PLOS Glob Public Health. 2024 Jun 27;4(6):e0001322. doi: 10.1371/journal.pgph.0001322 (PMC11210749; doi:10.1371/journal.pgph.0001322)
Supplement: S3 Text — (DOCX) [file pgph.0001322.s003.docx]

# S3 Appendix: Incidence and Mortality Results

## S3.1 Breast cancer incidence and mortality by geographical region

Table A displays the mean incidence over the period 2007-2018 and mortality over the period 2007-2018 for each of the 16 Chilean regions. Both are presented as age-adjusted and crude rates. They are sorted from north to south.

| Region | Region Name | Crude | Age adjusted | Crude | Age adjusted |
| --- | --- | --- | --- | --- | --- |
| XV | Arica y Parinacota | 68.8 | 50.9 | 14.1 | 10.0 |
| I | Tarapacá | 33.9 | 28.3 | 11.8 | 10.1 |
| II | Antofagasta | 50.0 | 41.0 | 11.8 | 9.9 |
| III | Atacama | 37.0 | 28.2 | 11.6 | 9.0 |
| IV | Coquimbo | 43.3 | 30.7 | 14.2 | 9.7 |
| V | Valparaíso | 70.5 | 45.5 | 19.6 | 11.7 |
| RM | Metropolitana de Santiago | 66.6 | 46.8 | 16.2 | 10.8 |
| VI | Libertador General Bernardo O’Higgins | 39.7 | 27.3 | 15.3 | 10.5 |
| VII | Maule | 48.6 | 33.8 | 13.7 | 9.3 |
| XVI | Ñuble | 56.6 | 37.8 | 17.2 | 10.9 |
| VIII | Biobío | 55.6 | 39.0 | 15.3 | 10.4 |
| IX | La Araucanía | 44.4 | 31.0 | 14.0 | 9.4 |
| XIV | Los Ríos | 62.5 | 42.7 | 13.4 | 8.9 |
| X | Los Lagos | 36.4 | 26.5 | 11.6 | 8.1 |
| XI | Aysén del General Carlos Ibáñez del Campo | 38.4 | 30.0 | 13.2 | 10.6 |
| XII | Magallanes y de la Antártica Chilena | 58.0 | 39.3 | 20.3 | 13.1 |

**Table A**: Average incidence and mortality (cases/100,000 women) over the period 2007-2018 by region.

## S3.2 Breast cancer incidence by age group

Table B presents age-specific incidence rates for each age interval for each year from 2007 to 2018.

| Age | 2007 | 2008 | 2009 | 2010 | 2011 | 2012 | 2013 | 2014 | 2015 | 2016 | 2017 | 2018 | Mean (Std) |
| --- | --- | --- | --- | --- | --- | --- | --- | --- | --- | --- | --- | --- | --- |
| 0-19 | 0.6 | 0.6 | 0.7 | 0.8 | 0.6 | 0.7 | 0.8 | 0.6 | 0.8 | 0.5 | 0.7 | 0.5 | 0.7 (0.1) |
| 20-24 | 2.0 | 3.2 | 3.8 | 2.3 | 3.3 | 3.0 | 3.8 | 2.0 | 2.2 | 3.5 | 4.5 | 3.1 | 3.1 (0.8) |
| 25-29 | 7.1 | 8.0 | 5.1 | 7.7 | 6.1 | 5.6 | 6.5 | 5.8 | 6.4 | 8.5 | 6.8 | 8.2 | 6.8 (1.0) |
| 30-34 | 11.6 | 12.1 | 14.0 | 14.0 | 13.0 | 18.9 | 16.0 | 15.2 | 17.0 | 20.5 | 17.8 | 17.9 | 15.7 (2.7) |
| 35-39 | 29.1 | 28.6 | 36.5 | 30.9 | 35.2 | 36.7 | 36.9 | 34.6 | 38.8 | 36.6 | 36.0 | 35.7 | 34.6 (3.1) |
| 40-44 | 60.5 | 61.2 | 66.9 | 59.7 | 67.1 | 74.4 | 72.9 | 68.1 | 70.5 | 80.3 | 72.1 | 65.2 | 68.2 (5.9) |
| 45-49 | 96.7 | 92.8 | 111.3 | 102.4 | 111.5 | 117.7 | 108.3 | 103.0 | 113.7 | 121.7 | 98.7 | 105.9 | 107.0 (8.3) |
| 50-54 | 104.2 | 108.1 | 125.1 | 113.5 | 113.7 | 125.0 | 122.2 | 110.0 | 133.0 | 130.7 | 108.5 | 118.0 | 117.7 (9.1) |
| 55-59 | 121.6 | 113.3 | 140.7 | 120.7 | 122.1 | 122.4 | 140.9 | 131.4 | 142.1 | 130.9 | 131.0 | 124.9 | 128.5 (8.9) |
| 60-64 | 150.6 | 156.5 | 164.7 | 160.0 | 161.1 | 164.7 | 158.3 | 146.3 | 142.9 | 143.7 | 146.6 | 138.9 | 152.9 (8.7) |
| 65-69 | 160.1 | 179.5 | 172.1 | 172.4 | 182.6 | 186.0 | 197.3 | 175.4 | 181.4 | 170.5 | 174.6 | 150.6 | 175.2 (11.5) |
| 70-74 | 147.3 | 145.8 | 175.1 | 181.8 | 177.8 | 179.8 | 187.7 | 195.8 | 197.0 | 188.1 | 187.8 | 186.8 | 179.2 (15.9) |
| 75-79 | 154.1 | 164.4 | 176.3 | 160.7 | 172.3 | 178.3 | 172.8 | 165.8 | 190.1 | 198.6 | 182.6 | 171.4 | 174.0 (11.9) |
| 80-84 | 178.6 | 163.7 | 164.9 | 189.2 | 168.8 | 163.1 | 179.7 | 172.2 | 165.8 | 168.2 | 148.5 | 143.6 | 167.2 (12.0) |
| 85+ | 235.9 | 236.0 | 228.9 | 206.2 | 209.6 | 172.6 | 178.4 | 171.4 | 147.7 | 165.6 | 171.2 | 107.0 | 185.9 (37.2) |

**Table B**: Crude incidence rate per year and age group (cases/100,000 women).

## S3.3 Breast cancer mortality by age group

Table C presents age-specific mortality rates for each age interval for each year from 2007 to 2018.

| Age | 2007 | 2008 | 2009 | 2010 | 2011 | 2012 | 2013 | 2014 | 2015 | 2016 | 2017 | 2018 | Mean (Std) |
| --- | --- | --- | --- | --- | --- | --- | --- | --- | --- | --- | --- | --- | --- |
| 0-19 | 0.0 | 0.0 | 0.0 | 0.0 | 0.0 | 0.0 | 0.0 | 0.0 | 0.0 | 0.0 | 0.0 | 0.0 | 0.0 (0.0) |
| 20-24 | 0.0 | 0.0 | 0.1 | 0.1 | 0.0 | 0.1 | 0.0 | 0.0 | 0.1 | 0.0 | 0.1 | 0.1 | 0.1 (0.1) |
| 25-29 | 0.5 | 1.2 | 0.9 | 0.9 | 1.1 | 0.3 | 1.2 | 0.9 | 0.4 | 0.5 | 0.3 | 1.0 | 0.8 (0.3) |
| 30-34 | 1.1 | 3.0 | 1.4 | 2.0 | 2.0 | 1.9 | 2.0 | 2.1 | 2.6 | 2.1 | 2.9 | 3.4 | 2.2 (0.6) |
| 35-39 | 4.6 | 5.3 | 5.5 | 6.0 | 4.0 | 4.9 | 5.0 | 7.0 | 6.2 | 4.8 | 4.1 | 5.2 | 5.2 (0.8) |
| 40-44 | 11.1 | 9.3 | 9.7 | 11.4 | 9.6 | 12.3 | 11.1 | 10.2 | 10.6 | 10.7 | 8.7 | 12.8 | 10.6 (1.2) |
| 45-49 | 18.8 | 16.6 | 19.6 | 18.9 | 17.2 | 17.2 | 19.7 | 17.7 | 17.5 | 17.3 | 14.8 | 14.1 | 17.4 (1.6) |
| 50-54 | 23.3 | 24.7 | 25.7 | 25.4 | 26.7 | 22.0 | 21.2 | 24.5 | 22.5 | 24.7 | 25.3 | 18.8 | 23.7 (2.2) |
| 55-59 | 33.4 | 29.0 | 32.2 | 31.0 | 29.0 | 29.6 | 28.9 | 27.8 | 32.8 | 30.0 | 29.7 | 29.5 | 30.3 (1.7) |
| 60-64 | 34.4 | 40.8 | 42.4 | 39.1 | 38.0 | 36.4 | 38.1 | 29.1 | 33.4 | 31.7 | 38.0 | 30.8 | 36.0 (4.0) |
| 65-69 | 45.4 | 48.0 | 53.2 | 47.8 | 48.0 | 44.9 | 43.3 | 46.4 | 47.6 | 47.2 | 38.7 | 39.7 | 45.8 (3.7) |
| 70-74 | 52.2 | 55.1 | 52.5 | 47.5 | 60.3 | 56.9 | 63.4 | 56.9 | 54.4 | 62.1 | 60.1 | 49.2 | 55.9 (4.8) |
| 75-79 | 60.8 | 72.6 | 70.7 | 69.0 | 55.6 | 68.9 | 61.3 | 66.3 | 74.3 | 69.1 | 71.6 | 83.2 | 68.6 (6.9) |
| 80-84 | 83.2 | 81.8 | 91.7 | 85.8 | 102.4 | 88.8 | 95.6 | 84.7 | 99.6 | 77.5 | 73.6 | 90.8 | 88.0 (8.3) |
| 85+ | 185.3 | 175.1 | 192.0 | 153.2 | 170.4 | 173.4 | 139.1 | 164.8 | 150.5 | 139.6 | 142.1 | 144.3 | 160.8 (17.7) |

**Table C**: Crude mortality rate by year and age group (cases/100,000 women).
