# Supplementary figures and images for: Breast cancer trends in Chile: Incidence and mortality rates (2007–2018)
Source: PLOS Glob Public Health. 2024 Jun 27;4(6):e0001322. doi: 10.1371/journal.pgph.0001322 (PMC11210749; doi:10.1371/journal.pgph.0001322)

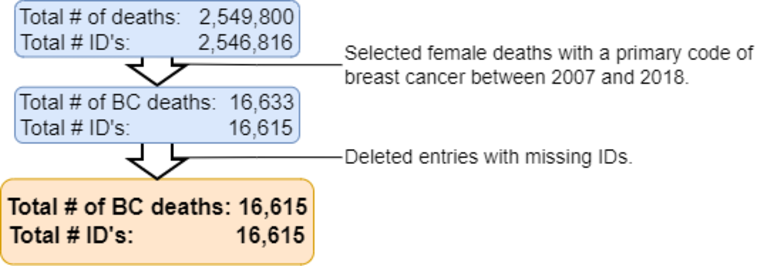

Supplement: S1 Fig — (TIF) [file pgph.0001322.s004.tif]

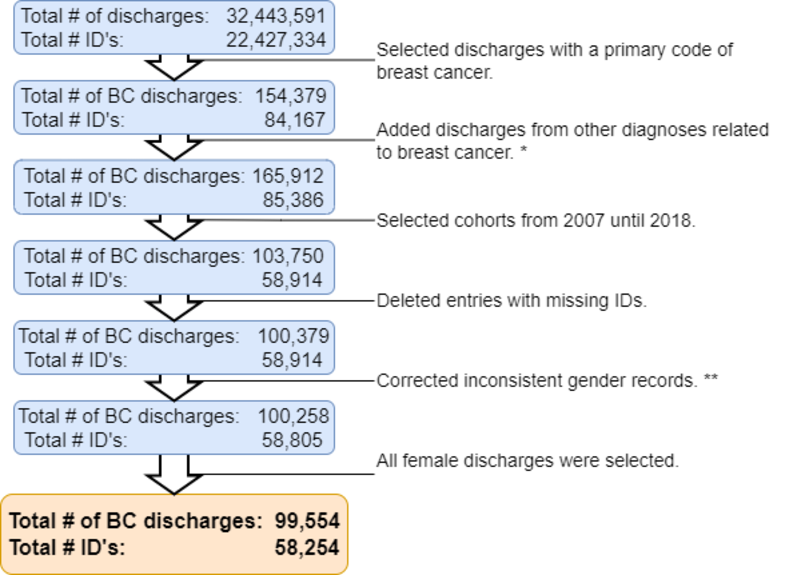

Supplement: S2 Fig — (TIF) [file pgph.0001322.s005.tif]
